# Association of Specialist Physician Payment Model With Visit Frequency, Quality, and Costs of Care for People With Chronic Disease

**DOI:** 10.1001/jamanetworkopen.2019.14861

**Published:** 2019-11-08

**Authors:** Amity E. Quinn, Brenda R. Hemmelgarn, Marcello Tonelli, Kerry A. McBrien, Alun Edwards, Peter Senior, Peter Faris, Flora Au, Zhihai Ma, Robert G. Weaver, Braden J. Manns

**Affiliations:** 1 Department of Community Health Sciences, Cumming School of Medicine, University of Calgary, Calgary, Alberta, Canada; 2 Department of Medicine, Cumming School of Medicine, University of Calgary, Calgary, Alberta, Canada; 3 Department of Family Medicine, Cumming School of Medicine, University of Calgary, Calgary, Alberta, Canada; 4 Department of Medicine, University of Alberta, Edmonton, Alberta, Canada; 5 Alberta Health Services, Calgary, Alberta, Canada

## Abstract

**Importance:**

Specialist physicians are key members of chronic care management teams; to date, however, little is known about the association between specialist payment models and outcomes for patients with chronic diseases.

**Objective:**

To examine the association of payment model with visit frequency, quality of care, and costs for patients with chronic diseases seen by specialists.

**Design, Setting, and Participants:**

A retrospective cohort study using propensity-score matching in patients seen by a specialist physician was conducted between April 1, 2011, and September 31, 2014. The study was completed on March 31, 2015, and data analysis was conducted from June 2017 to February 2018 and finalized in August 2019. In a population-based design, 109 839 adults with diabetes or chronic kidney disease newly referred to specialists were included. Because patients seen by independent salary-based and fee-for-service (FFS) specialists were significantly different in observed baseline characteristics, patients were matched 1:1 on demographic, illness, and physician characteristics.

**Exposures:**

Specialist physician payment model (salary-based or FFS).

**Main Outcomes and Measures:**

Follow-up outpatient visits, guideline-recommended care delivery, adverse events, and costs.

**Results:**

A total of 90 605 patients received care from FFS physicians and 19 234 received care from salary-based physicians. Before matching, the patients seen by salary-based physicians had more advanced chronic kidney disease (2630 of 14 414 [18.2%] vs 6627 of 54 489 [12.2%]), and a higher proportion had 5 or more comorbidities (5989 of 19 234 [31.3%] vs 23 326 of 90 605 [25.7%]). Propensity-score matching resulted in a cohort of 31 898 patients (15 949 FFS, 15 949 salary-based) seeing 489 specialists. In the matched cohort, patients were similar (mean [SD] age, 61.3 [18.2] years; 17 632 women [55.3%]; 29 251 residing in urban settings [91.7%]). Patients seen by salary-based specialists had a higher follow-up visit rate compared with those seen by FFS specialists (1.74 visits; 95% CI, 1.58-1.92 visits vs 1.54 visits; 95% CI, 1.41-1.68 visits), but the difference was not significant (rate ratio, 1.13; 95% CI, 0.99-1.28; *P* = .06). There was no statistical difference in guideline-recommended care delivery, hospital or emergency department visits for ambulatory care–sensitive conditions, or costs between patients seeing FFS and salary-based specialists. The median association of physician clustering with health care use and quality outcomes was consistently greater than the association with the physician payment, suggesting variation between physicians (eg, median rate ratio for follow-up outpatient visit rate was 1.74, which is greater than the rate ratio of 1.13).

**Conclusions and Relevance:**

Specialist physician payment does not appear to be associated with variation in visits, quality, and costs for outpatients with chronic diseases; however, there is variation in outcomes between physicians. This finding suggests the need to consider other strategies to reduce physician variation to improve the value of care and outcomes for people with chronic diseases.

## Key Points

**Question:**

Is a specialist physician payment model associated with visit frequency, quality of care, and costs for people with chronic disease?

**Findings:**

In this population-based cohort study that included a propensity-score matched cohort of 31 898 adults with diabetes or chronic kidney disease seen by 489 physicians, there was no statistical evidence of a difference in follow-up outpatient visit rates, quality, and costs between patients seeing salaried and fee-for-services physicians. The median association of physician clustering and the outcomes was greater than the association with the physician payment model.

**Meaning:**

Specialist physician payment does not appear to be associated with variation in use of chronic disease care, quality, and costs; however, these findings suggest large variation in outcomes between physicians.

## Teaser

This population-based cohort study examines the visit frequency, quality of care, and cost for patients with chronic diseases seen by fee-for-service vs salaried specialist physicians.

## Introduction

Noncommunicable chronic diseases pose a major challenge for health systems worldwide owing to rising prevalence and costs.^1^ Chronic disease management models have focused on the role of primary care^2^; however, specialists are also key members of the chronic care team providing additional support and care to patients with more complex needs.^3^ Outpatient care for chronic conditions is frequently suboptimal. There are effective interventions for chronic diseases,^4,5,6^ but less than half of eligible patients receive them.^7^ Physician payment has been identified as a barrier to the delivery of effective interventions for chronic disease.^8^

Fee-for-service (FFS) is the dominant physician compensation model in the United States and Canada: 95% of physician office visits in the United States^9^ and 72% of clinical payments in Canada^10^ were reimbursed under FFS. There is a robust literature on the association between payment mechanisms and physician behavior, but most of the empirical work addresses primary care payment and little is known about how specialists respond to payment models in general or, specifically, when caring for patients with chronic diseases. Studies in primary care found that FFS payment is associated with a higher number of primary care and specialty care visits compared with salary payments.^11,12^ Similarly, studies in specialty care have found FFS payment to be associated with increased health care use, including higher specialist visit rates^13^ and a higher volume of specialist physicians’ billable services, particularly for elective procedures.^14^ To date, few quasi-experimental studies of specialist payment have examined quality outcomes, and limited data exist on the outcomes associated with costs.^15^

Given the uncertainty around the association between the specialist physician payment model and care for patients with chronic diseases, we sought to examine the association of payment model with outpatient visit frequency, quality of care, and costs of care for patients with chronic diseases seen for the first time by a specialist by studying a payment reform in Alberta, Canada. The Academic Alternative Relationship Plan, offered to specialists in Calgary and Edmonton, Alberta, in 2004, compensated physicians with a salary-like payment that covered clinical, research, and teaching time. Physicians remained independent contractors and were not employees of Alberta’s health system. The goals were increased recruitment and retention, innovative care delivery, improved access to specialists, delivery of high-quality care, delivery of high-quality education, greater research capacity, and improved governance and accountability.^16^

## Methods

### Data Sources

We used the Interdisciplinary Chronic Disease Collaboration Data Repository.^17,18,19,20^ The database captures demographic, laboratory, and administrative health data (including vital statistics, prescription drug data, physician claims, hospitalizations, emergency department and outpatient visits, and all health care costs) for all individuals registered with Alberta Health (all residents of Alberta are eligible for insurance coverage; >99% participate^17^). Data are deidentified. This study was approved by University of Calgary’s Conjoint Health Research Ethics Board with waiver of informed consent. This study followed the Strengthening the Reporting of Observational Studies in Epidemiology (STROBE) reporting guideline.

### Study Cohort

The cohort included newly referred adults with preexisting diabetes or nondialysis chronic kidney disease (CKD) seen by a specialist physician (endocrinologist, nephrologist, or internal medicine physician) in Alberta between April 1, 2011, and September 30, 2014, with no visit to the same physician or a physician from the same specialty in the 4 years before their index visit.^21^ In Canada, internal medicine physicians deliver specialty care. We selected and combined these conditions because they represent a large proportion of patients with chronic disease, frequently co-occur, and share common risk factors and treatment guidelines. Only specialists practicing in Calgary and Edmonton were included because salary-based programs are only available in those cities.

**Figure 1.**
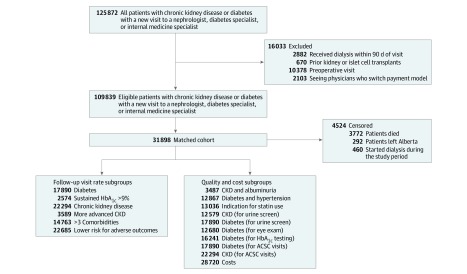
Cohort and Analysis Flow Diagram ACSC indicates ambulatory care–sensitive condition; CKD, chronic kidney disease; and HbA_1c_, hemoglobin A_1c_.

Patients with diabetes were identified using a validated algorithm.^22,23^ Chronic kidney disease was defined by serum creatinine level or—if serum creatinine level was not available—albuminuria or urine dipstick laboratory measurements before the index visit as in prior work.^24^ We excluded patients who (1) started dialysis within 90 days of their index visit, (2) had kidney or islet cell transplant before their index visit, (3) were seen for a preoperative visit (index visit within 60 days of an elective surgery^25,26^), and (4) saw a physician who switched payment models during the study period (Figure 1).

### Outcomes

#### Frequency of Visits

The primary outcome was the rate of outpatient visits with the primary specialist physician after the first visit. We included all patient visits (limited to 1 per day for estimation of rates) from physician claims data between the index date and the end of the study (March 31, 2015, unless a patient died, moved from Alberta, or started dialysis).

#### Quality Indicators

Secondary outcomes included the proportion of patients receiving guideline-recommended care and the rate of hospital admissions or emergency department visits for diabetes-specific, ambulatory care–sensitive conditions^27^ among patients with diabetes and for CKD-specific ambulatory care–sensitive conditions^28^ among those with CKD. Hospital visits were identified using hospitalization discharge data and emergency department visits were identified using ambulatory care data. Guideline-recommended care was defined using prescription drug data as the use of indicated medications 6 months after the index visit (angiotensin-converting enzyme inhibitors or angiotensin receptor blockers among people with CKD and albuminuria^29,30^ or diabetes and hypertension^31^; statins in patients aged >40 years with diabetes, those with diabetes and CKD, and in patients aged ≥50 years with CKD and no diabetes^32,33^) and using laboratory and claims data as the use of recommended testing following the index visit (urine albumin-creatinine ratio in those with CKD^30^ or diabetes; eye examinations and hemoglobin A_1c_ [HbA_1c _(SI conversion factor: To covert HbA1c to proportion of total hemoglobin, multiply by 0.01)] level for those with diabetes^34^). Only patients with follow-up time required for each measure were eligible to be included.

#### Costs

Mean total and categorical costs per patient for services that could potentially be associated with specialist physicians’ behavior (primary specialist, other specialist, and primary care physician claims) for hospitalization and emergency department visits for ambulatory care–sensitive conditions (CKD,^28^ chronic obstructive pulmonary disease, asthma, diabetes, heart failure, pulmonary edema, hypertension, angina^27^), and use of ambulatory care (diagnostic imaging, laboratory tests, and prescription drug costs) were estimated for the first year and second year after the index visit for patients with 1 and 2 full years of follow-up data. Costs were censored if a patient died, moved from Alberta, or started dialysis. Primary specialist physician costs were estimated using actual amounts paid to FFS physicians and shadow billing (ie, claims submitted for administrative purposes that do not result in direct reimbursement) estimates for salary-based physicians. All costs are reported in 2016 Canadian dollars (2016 exchange rate: $1.00 Canadian dollar = $0.75 US dollar).^35^

### Other Variables

The explanatory variable was the specialist physician payment model (salary-based vs FFS) at the patients’ index visit. A variable to consistently indicate payment model was added in 2011. Patient covariates included age, sex, neighborhood income quintile, rural vs urban residence status, prior use of health services, illness characteristics, and comorbidities defined using validated algorithms.^36^ Prior use of health services was defined as use of disease-related medications (ie, angiotensin-converting enzyme inhibitors, angiotensin receptor blockers, statins) in the 6 months before and hospitalizations and emergency department visits for ambulatory care–sensitive conditions in the year before the patient’s index visit. Illness characteristics included the presence of diabetes, CKD, or both conditions and details about the severity of their conditions based on laboratory measurements (ie, HbA_1c_, estimated glomerular filtration rate, albuminuria) and disease duration. Physician characteristics included physician type, defined as nephrologist, diabetes specialist (endocrinologists and internal medicine physicians with >50 different patients with diabetes each year and >30% of claims for outpatient diabetes care), and internal medicine physicians (excluding those in the diabetes specialist category); clinical workload (low, middle, and high based on the number of days billing per year); city of practice; and years practiced in Alberta since 1994.

### Statistical Analysis

We conducted the initial analysis from June 2017 to February 2018 and the final analysis in August 2019. We used propensity scores to adjust for baseline differences in patients and physicians. We first developed a multivariable logistic regression model including patient and physician characteristics to estimate the probability of being seen by a salary-based physician. Any missing data were defined as a category of variables included in the model (only 5 baseline characteristic variables had any missing data). We iteratively modified the patient and physician characteristics in the model if estimation and matching led to a sample with 1 or more characteristics with a standardized mean difference greater than or equal to 10. A standardized difference below 10% implied an acceptable balance.^37^ We matched 1 patient seen by an FFS physician with 1 patient seen by a salary-based physician (without replacement) on the closest matched propensity score (based on a caliper of 0.2 of the SD of the log odds of the propensity score).^37,38^ We assessed the balance in patient and physician characteristics between salary-based and FFS groups before and after matching using the standardized mean difference (expressed as a percentage). We further assessed the standardized differences in subgroup analyses (Figure 1).

Using the matched cohort, unadjusted overall visit rates were estimated using Poisson models for different physician characteristics by payment model. We examined the association between payment model and outcomes using generalized linear mixed models that included a fixed effect for payment model, a random intercept for a physician to address patients clustering within physicians, and a random residual for matched pairs to address correlation between propensity-matched pairs. We accounted for any imbalance (standardized difference ≥10%) in subgroups (Figure 1) by including unbalanced variables as covariates in mixed models. We assessed the association of payment model with visit rates and payment model with quality indicators by estimating rate ratios (RRs) using models of the Poisson family with a log link function, correcting for over-dispersion as necessary by using the negative binomial distribution.^39^ Follow-up visit rate and rate of hospital admissions and emergency department visits for diabetes- and CKD-specific ambulatory care–sensitive conditions mixed models included a denominator offset (ie, log number of days followed up) to account for patients’ variable follow-up times. Owing to the log-normal distribution of the random intercepts in the mixed models, FFS and salary-based rates and 95% CIs were transformed to population-averaged effects.^40,41^ The 95% CIs account for the variance of the fixed-effects estimates as well as the error in estimating the variance of the random intercepts. The association of mean total costs and payment model was estimated using the normal distribution.

For all use and quality outcomes, we also estimated median rate ratios (MRRs).^42^ The MRR is the median relative change in the outcome between a randomly selected patient seeing a physician with a higher rate of the outcome and a randomly selected patient with the same covariates seeing a physician with a lower rate of the outcome. The MRR quantifies the magnitude of the effect of clustering within physicians. An MRR greater than the RR for payment model suggests that the median association of physician clustering with an outcome was greater than the association with a payment model. An MRR lower than the RR suggests that the association of payment model with an outcome was greater than the median association with physician clustering.

For all outcomes, we addressed the issues of multiple comparisons by applying the Benjamini-Hochberg procedure^43^ that indicates which findings retain statistical significance after controlling for a prespecified false discovery rate. A false discovery rate of 10% was selected to balance our ability to detect the outcomes of interest against the consequence of false discoveries. Analysis was conducted using SAS statistical software, version 9.4 (SAS Institute Inc), and Stata statistical software, version 14.2 (StataCorp). The threshold for statistical significance was set at 2-tailed *P* < .05.

## Results

We excluded 2882 patients undergoing dialysis, 670 with kidney or islet cell transplants, 10 378 who had preoperative index visits, and 2103 whose physicians switched payment models during the study, resulting in a cohort of 109 839 adults with diabetes or nondialysis CKD newly referred to specialist physicians. There were 90 605 patients seen by FFS physicians and 19 234 seen by salary-based physicians. A higher proportion of patients with diabetes was seen by FFS physicians (65% vs 52%, *P* < .001), while a higher proportion of patients with CKD was seen by salary-based physicians (75% vs 60%, *P* < .001) (Table 1). In general, patients seen by salary-based physicians were sicker, with a higher proportion with more advanced CKD (2630 of 14 414 [18.2%] vs 6627 of 54 489 [12.2%]; *P* < .001) and a higher prevalence of 5 or more comorbidities (5989 of 19 234 [31.3%] vs 23 326 of 90 605 [25.7%]; *P* < .001), as well as a higher prevalence of chronic heart failure and dementia (Table 1; eTable 1 in the Supplement). Salary-based physicians were more likely than FFS physicians to be kidney specialists (standardized mean difference, 63%) and have a medium clinical workload (standardized mean difference, 90%) (Table 1).

**Table 1.** Characteristics of Outpatient Visits by Patients With Diabetes or CKD by Physician Payment Model, Before and After Matching by Propensity Score

Characteristic^a^	Before Matching			After Matching		
	No. (%)		Standardized Difference	No. (%)		Standardized Difference
	FFS Physician (n = 90 605)	Salary-Based Physician (n = 19 234)		FFS Physician (n = 15 949)	Salary-Based Physician (n = 15 949)	
**Demographic**						
Age, mean (SD), y	62.4 (15.9)	61.3 (18.7)	6.3	61.2 (17.8)	61.4 (18.7)	0.9
Men	46 909 (51.8)	8708 (45.3)	13.0	7067 (44.3)	7199 (45.1)	1.7
Women	43 696 (48.2)	10 526 (54.7)	13.0	8882 (55.7)	8750 (54.9)	1.7
Socioeconomic status						
Quintile 1 (lowest)	21 006 (23.2)	4353 (22.6)	1.3	3545 (22.2)	3579 (22.4)	0.5
Quintile 2	21 286 (24.5)	4280 (22.3)	3.0	3586 (22.5)	3567 (22.4)	0.3
Quintile 3	16 517 (18.2)	3325 (17.3)	2.5	3744 (17.2)	2762 (17.3)	0.3
Quintile 4	15 114 (16.7)	3237 (16.8)	0.4	2680 (16.8)	2688 (16.9)	0.1
Quintile 5 (highest)	14 720 (16.3)	3519 (18.3)	5.4	2926 (18.4)	2914 (18.3)	0.2
Urban residence	83 515 (92.2)	17 600 (91.5)	2.6	14 613 (91.6)	14 638 (91.8)	0.6
Primary care attachment^b^						
Infrequent	6425 (7.1)	1524 (7.9)	3.2	1202 (7.5)	1198 (7.5)	0.1
Low	12 055 (13.3)	2492 (13.0)	1.0	2195 (13.8)	2096 (13.1)	1.8
Medium	27 471 (30.3)	5714 (29.7)	1.3	4867 (30.5)	4789 (30.0)	1.1
High	44 654 (49.3)	9504 (49.4)	0.3	7685 (48.2)	7866 (49.3)	2.3
**Illness Severity**						
Diabetes	59 193 (65.3)	10 052 (52.3)	26.8	9053 (56.8)	8852 (55.5)	2.5
Baseline HbA_1c_, %			3.6			6.6
No.	54 463	9260		8291	8121	
Mean (SD)	7.7 (1.8)	7.6 (1.8)		7.7 (1.8)	7.7 (1.8)	
Proportion with sustained HbA_1c_>9%	7785 (13.2)	1409 (14.0)	2.5	1342 (14.8)	1233 (13.9)	0.7
Duration of diabetes, y			9.0			2.8
No.	59 193	10 052		9053	8852	
Mean (SD)	8.1 (5.8)	8.6 (5.9)		8.3 (5.9)	8.5 (5.9)	
CKD	54 489 (60.1)	14 414 (74.9)	32.0	11 027 (69.1)	11 279 (70.7)	3.5
Proportion with more advanced CKD^c^	6627 (12.2)	2630 (18.2)	17.0	1736 (15.7)	1854 (16.4)	1.9
Proportion with A2 or A3 albuminuria^c^	25 732 (47.3)	7228 (50.2)	5.9	5414 (49.1)	5577 (49.5)	0.7
eGFR, mL/min/1.73 m^2^			31.4			4.7
No.	28 468	8739		6192	6583	
Mean (SD)	46.8 (10.6)	43.2 (12.2)		44.8 (11.8)	44.3 (11.8)	
Both diabetes and CKD	23 077 (25.5)	5232 (27.2)	4.9	4131 (25.9)	4182 (26.2)	0.7
**Health Care Use **						
ACE inhibitor or ARB use in 6 mo before index visit, No. (%)						
Patients with eGFR 15-60 mL/min/1.73 m^2^ and moderate or severe albuminuria	4430 (69.6)	1904 (70.1)	1.2	1081 (67.5)	1340 (70.9)	7.4
Patients with diabetes and hypertension	33 216 (74.8)	5665 (75.4)	1.2	4795 (74.6)	4827 (74.9)	0.6
Statin use in 6 mo before index visit	41 226 (45.4)	7478 (38.9)	13.4	6275 (39.3)	6135 (38.5)	1.8
Admission to hospital or visits to EDs						
Diabetes-specific ACSC in year before visit in patients with diabetes, mean (SD)			9.1			1.1
No.	59 193	10 052		9053	8852	
Mean (SD)	0.3 (1.0)	0.4 (1.2)		0.4 (1.1)	0.4 (1.2)	
CKD-specific ACSC in year before visit in patients with CKD, mean (SD)			2.1			1.0
No.	54 489	14 414		11 027	11 279	
Mean (SD)	0.2 (0.7)	0.2 (0.7)		0.2 (0.7)	0.2 (0.7)	
Comorbidities^d^						
1	10 578 (11.7)	2241 (11.7)	0.1	2125 (13.3)	1852 (11.6)	5.2
2	20 682 (22.8)	3711 (19.3)	8.7	3319 (20.8)	3076 (19.3)	3.8
3-4	36 019 (39.8)	7293 (37.9)	3.8	5820 (36.5)	6119 (38.4)	3.9
≥5	23 326 (25.7)	5989 (31.3)	12.0	4685 (29.4)	4902 (30.7)	3.0
**Physician Characteristics**						
Practice type						
Nephrology	7213 (8.0)	6148 (32.0)	63.0	3020 (18.9)	3557 (22.3)	8.3
Diabetes specialist^e^	12 178 (13.4)	1585 (8.2)	16.8	1781 (11.2)	1573 (9.9)	4.3
Internal medicine	71 214 (78.6)	11 501 (59.8)	41.6	11 148 (69.9)	10 819 (67.8)	4.5
Clinical workload^f^						
Low	5047 (5.57)	2615 (13.6)	27.5	2495 (15.6)	2258 (14.2)	4.2
Medium	31 828 (35.1)	14 603 (75.9)	90.1	11 804 (74.0)	11 691 (73.3)	1.6
High	53 730 (59.3)	2016 (10.5)	119.3	1650 (10.4)	2000 (12.5)	6.9
Location						
Urban zone 1	34 871 (38.5)	11 842 (61.6)	47.4	9891 (62.0)	9803 (61.5)	1.1
Urban zone 2	55 734 (61.5)	7392 (38.4)	47.4	6058 (38.0)	6146 (38.5)	1.1
Years practicing in Alberta since 1994			24.0			0.9
No.	90 605	19 234		15 949	15 949	
Mean (SD)	11.3 (6.7)	9.8 (5.7)		10.1 (6.5)	10.0 (5.9)	

Abbreviations: ACE, angiotensin-converting enzyme; ARB, angiotensin receptor blocker; ACSC, ambulatory care–sensitive condition; CKD, chronic kidney disease; ED, emergency department; eGFR, estimated glomerular filtration rate; FFS, fee-for-service; HbA_1c_, hemoglobin A_1c_.

SI conversion factor: To covert HbA_1c_ to proportion of total hemoglobin, multiply by 0.01.

^a^ Most of the patient and physician characteristics had no missing values. Socioeconomic status had 2% (n = 2482) missing in the unmatched cohort and 3% (n = 907) in the matched cohort. Urban vs rural residency status had 0.14% (n = 2482) missing in the unmatched cohort and 0.1% (n = 33) missing in the matched cohort. Among those with diabetes, 8% were missing baseline HbA_1c_ measurements in the unmatched (n = 5522) and matched (n = 1493) cohorts. Among those with CKD, 46% were missing eGFR laboratory values in the unmatched cohort (n = 31696) and 43% were missing eGFR values in the matched cohort (n = 9531). Measures based on eGFR values (ie, ACE inhibitor and ARB use among people with specific eGFR values) would also have substantial missingness owing to missing eGFR values.

^b^ Primary care attachment is defined as infrequent (1-2 primary care visits), high (>75% of patients with ≥3 primary care visits made to the same physician), medium (50%-75% of ≥3 visits made to the same physician), and low (<50% of visits made to any 1 primary care physician).

^c^ More advanced CKD is defined as eGFR less than 30 mL/min/1.73 m^2^, eGFR less than 45 and moderate or severe albuminuria, or eGFR less than 60 mL/min/1.73 m^2^ with severe albuminuria. Moderate albuminuria is defined as albumin-creatinine ratio, 3 to 29 mg/mmol; protein-creatinine ratio, 15 to 49, urine dipstick albumin 1+. Severe albuminuria is defined as albumin-creatinine ratio 30 mg/mmol or more, protein-creatinine ratio 50 or more; and urine dipstick albumin 2+ or more.

^d^ Comorbidities include alcohol use disorder, asthma, atrial fibrillation, lymphoma, metastatic cancer, nonmetastatic cancer, chronic heart failure, chronic pain, chronic pulmonary disease, chronic viral hepatitis B, cirrhosis, dementia, depression, epilepsy, hypertension, hypothyroidism, inflammatory bowel disease, irritable bowel syndrome, multiple sclerosis, myocardial infarction, Parkinson disease, peptic ulcer disease, peripheral vascular disease, psoriasis, rheumatoid arthritis, schizophrenia, severe constipation, stroke or transient ischemic attack^36^ (for more details on specific comorbidities, see eTable 2 in the Supplement).

^e^ Diabetes specialists are endocrinologists and internal medicine physicians who see more than 50 patients with diabetes each year and more than 30% of claims are for outpatient diabetes care.

^f^ Clinical workload is defined as the following: low, less than 94 days of billing per year; medium, 95 to 221 days of billing per year; high, 222 to 365 days of billing per year.

Propensity-score matching resulted in a well-balanced matched sample (31 898 patients; 15 949 FFS, 15 949 salary-based physicians), with all standardized differences less than 10% (Table 1). In the matched cohort, mean (SD) age of patients seeing both types of specialists was 61.3 (18.2) years, 17 632 were women (55.3%), and 29 251 lived in urban areas (91.7%). A total of 489 physicians were included in the analysis.

### Follow-up Outpatient Visits

The unadjusted follow-up outpatient visit rates were 2.02 (95% CI, 1.69-2.40) for all patients seeing FFS specialists and 1.90 (95% CI, 1.67-2.15) for all patients seeing salary-based specialists (eTable 2 in the Supplement). Unadjusted follow-up visit rates varied by payment model and physician characteristics. The unadjusted follow-up visit rate was highest for patients seeing salary-based nephrologists (3.13; 95% CI, 3.07-319) and salary-based specialists with the highest clinical workload (3.16; 95% CI, 3.08-3.24), and the lowest rate was for patients seeing FFS specialists with the lowest clinical workload (0.98; 95% CI, 0.94-1.02).

After accounting for physician and matched pair clustering, patients seen by salary-based physicians had a higher rate of follow-up visits compared with those seen by FFS physicians (1.74; 95% CI, 1.58-1.92 vs 1.54; 95% CI, 1.41-1.68), but the difference was not significant (RR, 1.13; 95% CI, 0.99-1.28; *P* = .06) (Table 2). There was no statistical evidence of a difference in visit rates between specialist groups for any of the subgroups. The visit rate for patients at higher risk for adverse outcomes was greater compared with the rate for patients who were not at higher risk for both the FFS and salary-based specialist groups. For example, patients with sustained hemoglobin A_1c_ levels greater than 9% seeing FFS specialists had a visit rate of 3.09 (95% CI, 2.17-4.39), while those in the low-risk group seeing FFS specialists had a visit rate of 1.56 (95% CI, 1.42-1.71). The MRR was higher than the RR for all subgroups.

**Table 2.** Follow-up Visit Rate (per 1000 Patient-Days) of Patients With Diabetes or CKD Seen by Salary-Based and FFS Specialists^a^

Variable	FFS Physician		Salary-Based Physician		Patients of Salary-Based vs FFS Physicians		Randomly Selected Patients of Physicians With High vs Low No. of Visits, MRR
	No.	Rate (95% CI) per 1000 Patient-Days	No.	Rate (95% CI) per 1000 Patient-Days	Rate Ratio (95% CI)	*P* Value	
Overall	15939	1.54 (1.41-1.68)	15938	1.74 (1.58-1.92)	1.13 (0.99-1.28)	.06	1.74
All visits and procedures	15939	2.07 (1.90-2.25)	15938	2.27 (2.07-2.48)	1.09 (0.97-1.23)	.15	1.68
Diabetes	9047	2.05 (1.78-2.37)	8843	2.19 (1.87-2.55)	1.07 (0.87-1.30)	.53	2.37
Chronic kidney disease	11022	1.59 (1.44-1,74)	11272	1.81 (1.64-2.01)	1.14 (0.99-1.30)	.06	1.76
Patients at higher risk of adverse outcomes							
Sustained HbA_1c_ >9%	1342	3.09 (2.17-4.39)	1,232	2.61 (1.84-3.72)	0.85 (0.71-1.02)	.07	1.52
More advanced CKD^b^	1736	1.99 (1,42-2,80)	1853	2.09 (1.47-2.96)	1.05 (0.91-1.21)	.53	1.37
>3 Comorbidities	7103	2.31 (2.05-2.61)	7660	2.18 (2.00-2.66)	1.00 (0.87-1.15)	.97	1.69
Patients at lower risk of adverse outcomes							
<3 Comorbidities and HbA_1c_ <9% and less advanced CKD	11456	1.56 (1.42-1.71)	11229	1.71 (1.16-2.52)	1.10 (0.96-1.26)	.16	1.75

Abbreviations: CKD, chronic kidney disease; FFS, fee-for-service; HbA_1c_, hemoglobin A_1c_; MRR, median rate ratio.

SI conversion factor: To covert HbA1c to proportion of total hemglobin, multiply by 0.01.

^a^ Patients saw a total of 489 physicians (295 FFS, 194 salary-based). Censored for dialysis, death, and leaving Alberta. From April 1, 2011, to March 31, 2014 (n = 4224). A separate mixed model was run for each subgroup analysis. Payment model was the only variable (fixed effect) in the model unless there was imbalance in the subgroup, in which case the unbalanced variables were included in the model. Sustained HbA_1c_ greater than 9% adjusted for estimated glomerular filtration rate (categorical), number of comorbidities (categorical), physician zone, clinical workload, and years of billing (categorical). More advanced CKD adjusted for physician type, clinical workload, years of billing (categorical), and dementia. More than 3 comorbidities adjusted for physician type and location. The Benjamini-Hochberg procedure^43^ to control the false discovery rate was not applied because there was no statistical evidence of a difference in rates. Only 1 visit per day was included in the rate calculation, except for all visits and procedures rate, which does include multiple visits per day.

^b^ More advanced CKD is defined as estimated glomerular filtration rate less than 30 mL/min/1.73 m^2^, estimated glomerular filtration rate less than 45 and moderate or severe albuminuria, or estimated glomerular filtration rate less than 60 mL/min/1.73 m^2^ with severe albuminuria. Moderate albuminuria is defined as albumin-creatinine ratio, 3 to 29 mg/mmol; protein-creatinine ratio, 15 to 49, urine dipstick 1+. Severe albuminuria is defined as albumin-creatinine ratio 30 mg/mmol or more, protein-creatinine ratio 50 or more; and urine dipstick albumin 2+ or more.

### Guideline-Recommended Care

There were no clinically meaningful or significant differences in delivery of guideline-recommended prescribing or use of recommended testing for patients seeing salary-based physicians compared with patients seeing FFS physicians (Table 3). The proportion of patients receiving recommended medications in the 6 months after their index visit (Table 3) was higher than the proportion receiving those medications in the 6 months before their visit (Table 1). For example, before seeing a specialist, 9622 of 12 867 patients (74.8%) with diabetes and hypertension used an angiotensin-converting enzyme inhibitor or angiotensin receptor blocker, while in the 6 months after seeing an FFS specialists, 6426 patients (89.3%) (95% CI, 85.2-93.6) used one of these drugs and, in the 6 months after seeing a salary-based specialist, 6441 patients (85.1%) (95% CI, 81.1-89.3) did. The MRR was higher than the RR for 6 of the 7 guideline-recommended care outcomes.

**Table 3.** Delivery of Guideline-Recommended Care and Rates of Adverse Events for Patients With Diabetes or CKD Seen by Salary-Based and FFS Specialists^a^

Variable	FFS Physician		Salary-Based Physician		Patients of Salary-Based vs FFS Physicians		Randomly Selected Patients of Physicians With High vs Low No. Visits, MRR
	No.	Proportion of Exposed With Outcome, Rate % (95% CI)	No.	Proportion of Exposed With Outcome, Rate % (95% CI)	Rate Ratio (95% CI)	*P* Value	
**Guideline-Recommended Care**							
ACE inhibitor or ARB use in 6 mo following index visit							
Patients with eGFR 15-60 mL/min/1.73 m^2^ and moderate or severe albuminuria	1600	74 (62-87)	1887	74 (62-88)	1.01 (0.94-1.08)	.87	1.00
Patients with diabetes and hypertension	6426	89 (85-94)	6441	85 (81-89)	0.95 (0.89-1.01)	.13	1.23
Statin use in 6 mo following index visit in patients >40 y with diabetes, diabetes and eGFR 15-60 mL/min/1.73m^2^, and patients ≥50 y with eGFR 15-60 mL/min/1.73 m^2^ and no diabetes	6708	52 (46-60)	6328	51 (45-58)	0.98 (0.88-1.08)	.63	1.24
Urine albumin screening measured using ACR (3 mo before or 9 mo after index visits)							
Patients with eGFR 15-60 mL/min/1.73 mg^2^	6102	38 (32-45)	6477	39 (33-46)	1.02 (0.88-1.19)	.17	1.62
Annually in patients with diabetes	9047	59 (49-71)	8843	55 (45-66)	0.93 (0.85-1.01)	.09	1.33
Eye examination ≤2 y after index visit for patients with diabetes	6521	67 (63-70)	6159	65 (61-68)	0.97 (0.93-1.01)	.16	1.10
≥2 HbA_1c_ measurements in year after index visit for patients with diabetes	8248	69 (64-75)	7993	69 (64-75)	1.01 (0.97-1.06)	.52	1.15
**Adverse Events**							
Rate of admissions to hospital or ED visits (per 1000 patient days)							
For diabetes-specific ACSC in patients with diabetes after index visit	9047	1.47 (1.32-1.63)	8843	1.63 (1.47-1.81)	1.12 (0.96-1.29)	.15	1.48
For CKD-specific ACSC in patients with CKD after index visit	11 022	0.28 (0.25-0.32)	11 272	0.29 (0.26-0.33)	1.03 (0.87-1.22)	.75	1.53

Abbreviations: ACE, angiotensin-converting enzyme; ACSC, ambulatory care–sensitive condition; ARB, angiotensin receptor blockers; CKD, chronic kidney disease; ED, emergency department; eGFR, estimated glomerular filtration rate; FFS, fee-for-service; HbA_1c_, hemoglobin A_1c_; MRR, median rate ratio.

^a^ Patients saw a total of 489 physicians (295 FFS, 194 salary-based). For guideline-recommended care measures, only patients with full follow-up time required for each measure are included. A separate mixed model was run for each subgroup analysis. Payment model was the only variable (fixed effect) in the model unless there was imbalance in the subgroup, in which case the unbalanced variables were included in the model. The model for ACE inhibitor and ARB use among those with CKD and albuminuria adjusts for chronic disease category, physician type, years of billing (category), dementia, and stroke. The model for ACE inhibitor and ARB use among those with diabetes and hypertension adjusts for physician type. Statin use adjusts for years of billing category. Urine screening for patients with CKD adjusts for years of billing category. Eye examinations adjusts for physician type and years of billing category. HbA_1c_ measurements adjusts for eGFR, number of comorbidities (categorical), physician type, and clinical workload. The Benjamini-Hochberg^43^ procedure to control the false discovery rate was not applied because there was no statistical evidence of a difference in rates.

### Hospital Admissions or Emergency Department Visits

For patients with diabetes, the rate of visits to the hospital or emergency department for diabetes-specific ambulatory care–sensitive conditions showed no statistical evidence of a difference among patients seeing salary-based compared with FFS physicians (RR, 1.12; 95% CI, 0.96-1.29) (Table 3). There did not appear to be a difference in the rate of visits to hospitals or emergency departments for CKD-specific ambulatory care–sensitive conditions between salary-based and FFS physicians for people with CKD (RR, 1.03; 95% CI, 0.87-1.22). The MRR was higher than the RR for both diabetes- and CKD-specific ambulatory care–sensitive condition visit rates.

### Costs

**Figure 2.**
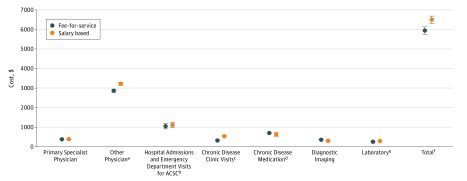
Unadjusted Mean Total and Categorical Costs per Patient (2016 CAD$) in the Year After Patient's Index Visit by Physician Payment Model Costs are reported in 2016 Canadian dollars (2016 exchange rate: $1.00 Canadian dollar = $0.75 US dollar). ACSC indicates ambulatory care–sensitive condition. ^a^Includes other specialists and primary care physician costs. ^b^Ambulatory care–sensitive conditions include chronic kidney disease–specific ACSCs^27^ and the following Canadian Institutes for Health Information–defined conditions: chronic obstructive pulmonary disease, asthma, diabetes, heart failure and pulmonary edema, hypertension, and angina.^26^ ^c^Includes nephrology, cardiology, and diabetes clinics. ^d^Chronic disease medications include antiarrhythmic drugs, nitrates and nitrites, statins, nonstatin cholesterol-lowering drugs, β-blockers, angiotensin-converting enzyme inhibitors, angiotensin receptor blockers, calcium channel blockers, diuretics, other blood pressure–lowering medications, anticoagulants, antidiabetes medications, antiplatelet agents, insulin, smoking cessation aids, erythropoietin, and darbepoetin alfa. ^e^Includes the 25 of the most frequently ordered diagnostic tests at Canadian laboratories: complete blood cell count; prothrombin time (international normalized ratio); and creatinine, alanine aminotransferase, thyroid-stimulating hormone, hemoglobin A_1c_, low-density lipoprotein cholesterol, ferritin, alkaline phosphatase, albumin, random glucose, fasting glucose, calcium, urea, magnesium, iron and total iron-binding capacity, phosphate, total bilirubin, creatine kinase, free thyroxine, prostate-specific antigen, urate, lactate dehydrogenase, lipase, and albumin random urine levels. ^f^Total is the sum of all categorical costs.

There was variation in unadjusted categorical costs by payment model (Figure 2; eTable 3 in the Supplement). Chronic disease medication ($701; 95% CI, $674-$727 vs $628; 95% CI, $606-$727) and diagnostic imaging ($359; 95% CI, $351-$368 vs $302; 95% CI, $294-$309) costs in the first year after index visits were higher for patients seeing FFS specialists compared with those seeing salary-based specialists; all other categorical costs were lower. The adjusted mean total costs per patient in the year after the index visit were not significantly different for patients seeing FFS compared with salary-based specialists ($6916; 95% CI, $6482-$7352 vs $6925; 95% CI, $6469-$7380; *P* = .98). Mean total and categorical costs per patient were lower in the second year compared with the first year after the index visit. For example, among patients seeing salary-based specialists, diagnostic imaging was $302 (95% CI, $294-$309) in the first year and $177 (95% CI, $171-$183) in the second year after the index visit (eTable 3 in the Supplement).

## Discussion

In this study of specialist physician payment for chronic disease care, we found no statistical difference in follow-up outpatient visit rates, quality of care, or costs between patients seen by salary-based and FFS physicians. The MRR was larger than the RR for most follow-up visit rates and quality outcomes, suggesting that the median association with physician clustering was greater than the association with the physician payment model. This finding suggests variation in care among physicians.

Patients seeing salary-based physicians had a higher number of outpatient visits than patients seeing FFS physicians, which is not consistent with primary care research.^11,12^ However, previous research in specialty care payment also found no significant difference in the number of outpatient visits to salaried and FFS specialists.^44^ It is possible the salary-based payment model in Alberta may influence other types of health care use (eg, other patient groups or inpatient settings). Research comparing the association between specialist salary and FFS payment models with use in other health care settings found that salary payment led to a significant decrease in elective tubal ligation,^44^ but there was no statistical evidence of a difference in emergency department use,^45^ inpatient anesthesia use,^46^ or number of surgical procedures.^44^

In primary care settings, salary-based payment is associated with poorer quality of care in terms of continuity and adherence to the recommended number of visits compared with FFS payment; however, patients seeing salaried physicians report higher satisfaction.^11,12^ To our knowledge, no quasi-experimental studies comparing salary and FFS payment in specialty care have examined quality or cost outcomes. Although we found no statistical evidence of a difference in quality between FFS and salary-based specialists, the proportion of patients receiving recommended medications was higher after their first specialist visit compared with the year before the visit, which suggests that specialists are important partners in caring for people with chronic diseases regardless of the payment model.

Given that it is unlikely there will be randomized clinical trials of specialist payment models, we believe it is important to consider differences in patients selected by physicians and differences in the physicians who select different payment models when determining the outcome and implications of physician payment reforms. In contrast to the theoretical literature suggesting that FFS specialists select sicker patients,^47^ we found that patients seen by salary-based physicians were sicker, pointing to possible differences in the behavior of specialists selecting patients compared with primary care physicians. Results of a survey of 135 physicians participating in Alberta’s Academic Alternative Relationship Plan indicated that most believed that the payment reform had a positive association with their ability to spend more time with patients with complex needs.^48^ Salary-based physicians seeing sicker patients is an important outcome of the payment reform and points to the relevance of a salary-based model for specialists seeing patients with chronic diseases and participating in chronic disease management teams.

Our results suggest that individual physician practice behaviors have a greater association than payment model with use of outpatient services and quality outcomes. Because physicians in this study selected their payment model, it is possible that there is an association between payment model and physician characteristics in practice variation. For example, female specialist physicians are more likely to select alternative payment models^12^ and also more likely to deliver higher-quality diabetes care than male specialists.^49^ Research identifying significant associations between payment models and use, quality, and cost outcomes does not typically account for physician clustering. A randomized clinical trial of physician incentives that used mixed models to account for physician clustering also found no effect of physician incentives, although the trial identified an effect of combined physician and patient incentives.^50^ We believe it would be prudent to address physician clustering, physician characteristics that may be associated with practice variation, and other contextual factors, such as practice site, in future research on physician payment models in specialty and primary care to expand our understanding of the differences in physician behavior.

### Limitations

Our study has a number of limitations. First, while the inclusion of patients with diabetes and CKD is clinically relevant and the propensity matching increases the internal validity of the results, the generalizability outside of nephrology, endocrinology, and internal medicine physicians in Alberta is uncertain. Second, although we used propensity-score matching to control for measured patient differences, it is possible that there were unmeasured differences even in our matched sample. Third, salary-based physicians are more likely to practice at tertiary care hospitals and in major urban centers than are FFS physicians,^51^ which might influence case mix and illness severity. Fourth, it is possible that some of the differences that we observed related to the accuracy of shadow billing, but a past analysis of our data comparing shadow and FFS billing with medical record reviews showed similar accuracy.^52^

## Conclusions

In this study, specialist physician payment is not associated with variation in chronic disease care outpatient visits, quality, and costs; however, we found a large variation in these outcomes among physicians. This variation suggests the need to consider other strategies, including incorporating principles of behavioral economics into payment models,^53^ to reduce variation between physicians to improve the value of care and outcomes for people with chronic diseases.

## Notes

Supplement. **eTable 1.** Comorbidity Characteristics of Patients With Diabetes or Chronic Kidney Disease by Physician Payment Model, Before and After Matching by Propensity Score **eTable 2.** Unadjusted Overall Visit Rate for the Matched Cohort by Specialist Physician Characteristics and Payment Model **eTable 3.** Unadjusted Mean Total and Categorical Costs per Patient (2016 CAD$) of Matched Cohort, One and Two Years After Index Visit by Primary Specialist Payment Model **eReferences**
