# Supplementary material for: Association of Specialist Physician Payment Model With Visit Frequency, Quality, and Costs of Care for People With Chronic Disease
Source: JAMA Netw Open. 2019 Nov 8;2(11):e1914861. doi: 10.1001/jamanetworkopen.2019.14861 (PMC6902778; doi:10.1001/jamanetworkopen.2019.14861)
Supplement: Supplement. — eTable 1. Comorbidity Characteristics of Patients With Diabetes or Chronic Kidney Disease by Physician Payment Model, Before and After Matching by Propensity Score eTable 2. Unadjusted Overall Visit Rate for the Matched Cohort by Specialist Physician Characteristics and Payment Model eTable 3. Unadjusted Mean Total and Categorical Costs per Patient (2016 CAD$) of Matched Cohort, One and Two Years After Index Visit by Primary Specialist Payment Model eReferences [file jamanetwopen-2-e1914861-s001.pdf]

Supplementary Online Content

Quinn AE, Hemmelgarn BR, Tonelli M, et al. Association of specialist physician payment model with visit frequency, quality, and costs of care for people with chronic disease. *JAMA Netw Open*. 2019;2(11): e1914861. doi:10.1001/jamanetworkopen.2019.14861

eTable 1. Comorbidity Characteristics of Patients With Diabetes or Chronic Kidney Disease by Physician Payment Model, Before and After Matching by Propensity Score

eTable 2. Unadjusted Overall Visit Rate for the Matched Cohort by Specialist Physician Characteristics and Payment Model

eTable 3. Unadjusted Mean Total and Categorical Costs per Patient (2016 CAD$) of Matched Cohort, One and Two Years After Index Visit by Primary Specialist Payment Model

eReferences

This supplementary material has been provided by the authors to give readers additional information about their work.

© 2019 Quinn AE et al. *JAMA Network Open.*

eTable 1. Comorbidity characteristics of patients with diabetes or chronic kidney disease by physician payment model, before and after matching by propensity score

	Before matching					After matching				
	Fee-for-service		Salary-based		Standardized difference	Fee-for-service		Salary-based		Standardized difference
	N=90605		N=19234			N=15949		N=15949		
	n	%	n	%		n	%	n	%	
Alcohol use disorder	3931	4.3	986	5.1	3.7	799	5.0	788	4.9	0.3
Asthma	5080	5.6	1153	6.0	1.7	973	6.1	984	6.2	0.3
Atrial Fibrilation	9451	10.4	2320	12.1	5.2	1843	11.6	1934	12.1	1.8
Cancer, lymphoma	956	1.1	237	1.2	1.7	208	1.3	191	1.2	1.0
Cancer, metastatic	1256	1.4	383	2.0	4.7	317	2.0	305	1.9	0.5
Cancer, non metastatic*	4776	5.3	1138	5.9	2.8	935	5.9	938	5.9	0.1
Chronic heart failure	11173	12.3	3047	15.8	10.1	2342	14.7	2450	15.4	1.9
Chronic pain	12207	13.5	3056	15.9	6.8	2593	16.3	2594	16.3	0.0
Chronic pulmonary disease	21018	23.2	4334	22.5	1.6	3560	22.3	3616	22.7	0.8
Chronic viral hepatitis B	173	0.2	197	1.0	10.7	93	0.6	100	0.6	0.6
Cirrhosis	569	0.6	330	1.7	10.1	215	1.4	220	1.4	0.3
Dementia	3119	3.4	1527	7.9	19.5	1154	7.2	1169	7.3	0.4
Depression	11048	12.2	2626	13.7	4.4	2290	14.4	2225	14.0	1.2
Epilepsy	2081	2.3	583	3.0	4.6	480	3.0	481	3.0	0.0
Hypertension	65496	72.3	13529	70.3	4.3	10803	67.7	11132	69.8	4.5
Hypothyroidism	14998	16.6	2279	17.6	2.7	2824	17.7	2848	17.9	0.4
Inflammatory bowel disease	1568	1.7	379	2.0	1.8	299	1.9	317	2.0	0.8
Irritable bowel syndrome	3231	3.6	627	3.3	1.7	516	3.2	542	3.4	0.9
Multiple sclerosis	775	0.9	177	0.9	0.7	142	0.9	143	0.9	0.1
Myocardial infarction	6760	7.5	1146	6.0	6.0	964	6.0	965	6.1	0.0
Parkinson's disease	618	0.7	163	0.9	1.9	128	0.8	129	0.8	0.1
Peptic ulcer disease	358	0.4	102	0.5	2.0	78	0.5	80	0.5	0.2
Peripheral vascular disease	3171	3.5	797	4.1	3.4	633	4.0	644	4.0	0.4
Psoriasis	1239	1.4	206	1.1	2.7	183	1.2	172	1.1	0.7
Rheumatoid arthritis	4506	5.0	1604	8.3	13.5	1283	8.0	1284	8.1	0.0

© 2019 Quinn AE et al. *JAMA Network Open.*

Schizophrenia	1675	1.9	306	1.6	2.0	1284	8.1	251	1.6	0.8
Severe constipation	2325	2.6	640	3.3	4.5	509	3.2	518	3.3	0.3
Stroke or TIA	12646	14.0	3027	15.7	5.0	2398	15.0	2511	15.7	2.0

* includes breast, cervical, colorectal, lung, prostate cancer

© 2019 Quinn AE et al. *JAMA Network Open.*

eTable 2. Unadjusted overall visit rate for the matched cohort by specialist physician characteristics and payment model

	N	FFS	95% CI	ARP	95% CI
**Overall**	31877	2.02	(1.69, 2.40)	1.90	(1.67, 2.15)
**Provider Type**[a]					
Kidney specialist	6575	3.07	(3.01, 3.14)	3.13	(3.07, 3.19)
Diabetes specialist	3349	2.25	(2.18, 2.31)	1.97	(1.90, 2.04)
Internal Med	21953	1.63	(1.63, 1.68)	1.44	(1.41, 1.46)
**Clinical Workload**[b]					
Low	4751	0.977	(0.94, 1.02)	1.29	(1.24, 1.34)
Medium	23478	2.12	(2.09, 2.15)	1.79	(1.76, 1.81)
High	3648	2.84	(2.76, 2.93)	3.16	(3.08, 3.24)
**Years Billing in Alberta since 1994**					
<5	6943	1.71	(1.66, 1.77)	1.49	(1.44, 1.53)
5 to 10	11194	1.79	(1.75, 1.83)	1.96	(1.92, 2.00)
11 to 17	9315	2.53	(2.48, 2.57)	2.17	(2.13, 2.22)
18+	4425	1.6	(1.53, 1.66)	1.68	(1.62, 1.75)

Patients saw a total of 489 physicians (295 FFS, 194 salary-based). Censored for dialysis, death, and leaving Alberta. From April 1, 2011-March 31, 2014 (N=4224)
[a]Kidney specialists are all nephrologists; diabetes specialists are endocrinologist and internal medicine physicians who see >50 patients with diabetes each year and >30% of claims are for outpatient diabetes care [b]Clinical workload is defined as the following: low=less than 94 days billing per year, medium=95-221 days billing per year, high=222-365 days billing per year.

© 2019 Quinn AE et al. *JAMA Network Open.*

eTable 3. Unadjusted mean total and categorical costs per patient (2016 CAD$) of matched cohort, one and two years after index visit by primary specialist payment model

	First year after index visit[a]				Second year after index visit[b]			
	Fee-for-service (n=14365)		Salary-based (n=14355)		Fee-for-service (n=13164)		Salary-based (n=13235)	
	Mean cost	95% confidence interval	Mean cost	95% confidence interval	Mean cost	95% confidence interval	Mean cost	95% confidence interval
Primary specialist physician	384	(378 - 389)	399	(394 - 404)	68	(66 - 70)	75	(72 - 78)
Other specialist physician	1897	(1835 - 1960)	2242	(2172 - 2312)	1190	(1144 - 1236)	1299	(1248 - 1349)
Primary care physician	962	(940 - 984)	976	(953 - 998)	720	(700 - 739)	718	(700 - 736)
Hospital admission for ACSCs[c]	974	(838 - 1110)	1045	(921 - 1168)	606	(503 - 709)	668	(573 - 763)
Emergency visits for ACSCs[c]	73	(66 - 80)	77	(70 - 83)	50	(46 - 55)	59	(53 - 64)
Nephrology clinics	111	(99 - 122)	195	(176 - 215)	87	(78 - 96)	156	(135 - 178)
Cardiology clinics	195	(177 - 212)	294	(270 - 319)	101	(89 - 123)	154	(137 - 172)
Diabetes clinics	25	(22 - 28)	45	(42 - 49)	7	(6 - 9)	12	(11 - 14)
Chronic disease medication[d]	701	(674 - 727)	628	(606 - 727)	621	(590 - 652)	565	(539 - 591)
Diagnostic imaging	359	(351 - 368)	302	(294 - 309)	192	(186 - 199)	177	(171 - 183)
Laboratory[e]	261	(254 - 268)	295	(288 - 302)	187	(181 - 192)	207	(201 - 213)
Total	5941	(5752 - 6130)	6498	(6315 - 6681)	3830	(3685 - 3974)	4091	(3947 - 4233)

Patients saw a total of 489 physicians (295 FFS, 194 salary-based). Censored for dialysis, death, and leaving Alberta. From April 1, 2011-March 31, 2014 (N=4224). [a]includes costs for patients with index visit dates April 1, 2011-March 31, 2014 to ensure all patients have one year of follow up data; [b]includes costs for patients with index visit dates April 1, 2011-March 31, 2013 to ensure all patients have two years of follow-up data; [c]ACSCs (Ambulatory Care Sensitive Conditions) include CKD-specific ACSCs [1] and the following CIHI defined conditions: chronic obstructive pulmonary disease, asthma, diabetes, heart failure and pulmonary edema, hypertension, and angina [2]. [d]Chronic disease medications include antiarrhythmic drugs, nitrates and nitrites, statins, non-statin cholesterol lowering drugs, beta blockers, ACE-inhibitors, angiotensin receptor blockers, calcium channel blockers, diuretics, other blood pressure medications, anticoagulants, anti-diabetes medications, anti-platelet agents, insulin, smoking cessation aids, erythropoietin, and darbepoietin; [e]includes 25 of the most frequently ordered diagnostic tests at Canadian laboratories: completed blood count, creatinine, ALT, thyroid stimulating hormone, hemoglobin A1c, LDL cholesterol, ferritin, alkaline phosphate, prothrombin time (INR), albumin, glucose random, glucose fasting, calcium, urea, magnesium, iron and TIBC, phosphate, Bilirubin total, creatine kinase, free T4, prostate specific antigen, urate, lactate dehydrogenase, lipase, albumin random urine; [f]Total is the sum of all categorical costs.

© 2019 Quinn AE et al. *JAMA Network Open.*

eReferences

1. Gao S, Manns BJ, Culleton BF, et al. Access to health care among status Aboriginal people with chronic kidney disease. *Canadian Medical Association Journal.* 2008;179(10):1007-1012.
2. Canadian Institute for Health Information (CIHI). Technical note: ambulatory care sensitive conditions (ACSC). 2010; https://www.cihi.ca/en/phc_policy_acsc_en.pdf. Accessed January 31, 2018, 2018.

© 2019 Quinn AE et al. *JAMA Network Open.*
